# Patient-reported outcomes for patients with breast cancer undergoing radiotherapy: A single-center registry experience

**DOI:** 10.3389/fonc.2022.920739

**Published:** 2022-08-26

**Authors:** Brady S. Laughlin, Ronik S. Bhangoo, Cameron S. Thorpe, Michael A. Golafshar, Todd A. DeWees, Justin D. Anderson, Tamara Z. Vern-Gross, Lisa A. McGee, William W. Wong, Michele Y. Halyard, Sameer R. Keole, Carlos E. Vargas

**Affiliations:** ^1^ Department of Radiation Oncology, Mayo Clinic, Phoenix, AZ, United States; ^2^ Department of Health Sciences Research, Mayo Clinic, Scottsdale, AZ, United States

**Keywords:** radiotherapy, breast cancer, patient-reported outcomes, CTCAE, survey

## Abstract

**Background:**

We present Patient-Reported Outcomes Version of the Common Terminology Criteria for Adverse Events (PRO-CTCAE) for patients undergoing adjuvant radiotherapy for breast cancer with curative intent. We describe the frequency and severity of PRO-CTCAE and analyze them with respect to dose fractionation.

**Methods:**

Patients were included in this study if they were treated with curative intent for breast cancer and enrolled on a prospective registry. Patients must have completed at least one baseline and one post-radiation survey that addressed PRO-CTCAE. For univariate and multivariate analysis, categorical variables were analyzed by Fisher’s exact test and continuous variables by Wilcoxon rank sum test. PRO-CTCAE items graded ≥2 and ≥3 were analyzed between patients who received hypofractionation (HF) versus standard conventional fractionation (CF) therapy by the Chi-square test.

**Results:**

Three hundred thirty-one patients met inclusion criteria. Pathologic tumor stage was T1–T2 in 309 (94%) patients. Eighty-seven (29%) patients were node positive. Two hundred forty-seven patients (75%) experienced any PRO-CTCAE grade ≥2, and 92 (28%) patients experienced any PRO-CTCAE grade ≥3. CF was found to be associated with an increased risk of grade ≥3 skin toxicity, swallowing, and nausea (all p < 0.01). HF (OR 0.48, p < 0.01) was significant in the multivariate model for decreased risk of any occurrence of PRO-CTCAE ≥3.

**Conclusions:**

Our study reports one of the first clinical experiences utilizing multiple PRO-CTCAE items for patients with breast cancer undergoing radiation therapy with curative intent. Compared with CF, HF was associated with a significant decrease in any PRO-CTCAE ≥3 after multivariate analysis.

## Introduction

In the setting of clinical cancer trials, physicians have been traditionally responsible for grading and recording patient adverse events according to the US National Cancer Institute’s (NCI) Common Terminology Criteria for Adverse Events (CTCAE). However, studies have shown that physician ratings of adverse events may not be accurate and that patient self-reporting of their side effects may provide complementary information to the provider’s determinations (1–3). Interest in a standardized approach for patient-reported adverse events led to the creation of the NCI’s Patient-Reported Outcomes Version of the CTCAE (PRO-CTCAE) (4). PRO-CTCAE items address three areas: frequency, severity, and interference with daily life. Individual items were initially adapted from CTCAE symptoms amenable to self-reporting.

PRO-CTCAE has been demonstrated to be valid and reliable against other quality-of-life measures across multiple malignancies (5). For patients undergoing radiation, site-specific PRO-CTCAE questionnaires were shown to be valid in assessing symptomatic toxicities (6). Feasibility of employing PRO-CTCAE has also been reported both at an institutional level and for a prospective clinical trial (7–9). NRG Oncology RTOG 1203 (comparing intensity modulated radiation therapy (IMRT) with standard four-field radiotherapy for gynecologic cancers) revealed a significant decrease in GI toxicities for IMRT patients when using PRO-CTCAE but did not reveal a corresponding difference in physician-reported CTCAE (10).

As discussed above, much of the evidence for PRO-CTCAE has focused on reliability, validity, and feasibility. Studies reporting PRO-CTCAE for patients undergoing radiation for a specific cancer are limited. Given the incidence of breast cancer and that radiotherapy is a common component of its treatment regimen, evaluating PRO-CTCAE in the context of breast cancer treatment would be particularly useful.

This study was designed with the intent to capture patient reported toxicities in patients with breast cancer. We present an initial clinical experience with PRO-CTCAE for patients undergoing adjuvant radiotherapy for breast cancer with curative intent. We describe the frequency and severity of adverse events using PRO-CTCAE in this population and analyze them with respect to clinical and radiotherapy characteristics.

## Methods

### Study design, patient selection, and survey administration

Patients at a single institution were eligible if they were enrolled with the Proton Collaborative Group (PCG; REG001-09, NCT01255748, informed consent obtained), which is a prospective multi-institutional observational study for patients treated at proton therapy centers within the United States. Institutional Review Board (IRB 19-000891) was also obtained.

Patients were included in this study if they were treated with curative intent for breast cancer using either photon or proton therapy between March 2016 and June 2019. Patients with metastatic disease were eligible if the treating physician determined the radiation therapy to be curative. All curative dosing fractionations were allowed. Patients undergoing whole breast, chest wall, or accelerated partial breast irradiation (APBI) were included for analysis. Technique to address lymph nodes at the time of surgery was not required for this study. Patients could have received systemic therapy as part of their treatment regimen at any time point.

Patients must have completed at least one baseline and one post-radiation survey which addressed PRO-CTCAE; otherwise, they were excluded from this analysis. In our department, patient surveys are routinely administered before, during, and after completion of radiotherapy (baseline, end of treatment, 3 months, 6 months, 12 months, and annually thereafter). We utilize the Patient-Reported Outcomes Measurement Information System 10 (PROMIS-10), which has been validated across multiple medical specialties (11, 12). Additional surveys administered include the Expanded Prostate Cancer Index breast-specific questions and an institution-specific PRO survey. Patients are not required to complete every survey provided to them.

### Radiotherapy

Radiation technique (proton or photon), dose fractionation, lumpectomy boost, lymph node coverage, and target volumes were all determined by the treating physician and treatment planning team. Bolus was not used for any patients. Organ-at-risk (OAR) dose constraints generally followed the institutional guidelines. Hypofractionation (HF) was defined as a dose per fraction >2 Gy. Proton therapy was delivered by a Hitachi pencil beam scanning treatment deliver system.

### Data collection

Patient, tumor, and treatment characteristics were collected from the PCG database. When necessary, additional data were obtained from electronic medical record system. As described above, three surveys related to patient-reported outcomes and quality-of-life measures are routinely administered to patients in our department. From these surveys, 14 symptom-related questions were selected to be included for evaluation as they reflected PRO-CTCAE items. Symptoms included anxiety, appetite, concentration, constipation, cough, insomnia, interference with breathing, nausea, sadness, severity of breathing interference, skin toxicity, stools (diarrhea), swallowing, and tingling. Each item is scored on a five-point Likert scale with regard to frequency, severity, and interference. Physician reported toxicities were also gathered using CTCAE version 4.0. Toxicities were considered acute or late whether they occurred before or after 3 months following adjuvant radiotherapy, respectively.

### Statistical analysis

Descriptive statistics were used to report patient, tumor, treatment, and PRO-CTCAE characteristics. For univariate and multivariate analysis, categorical variables were analyzed by Fisher’s exact test and continuous variables by Wilcoxon rank sum test. PRO-CTCAE items graded ≥2 and ≥3 were specifically analyzed between patients who received HF versus standard fraction therapy by Chi-square test.

## Results

### Patient and tumor characteristics

Three hundred thirty-one patients met inclusion criteria, 246 of which underwent HF and 85 underwent (CF). Baseline patient and tumor characteristics between patients treated with HF vs. CF are listed in **Table 1**. The median age at radiation therapy (RT) start was 60.0 years (62 HF vs. 54 CF). Most patients were Caucasian (312, 95%) with an Eastern Cooperative Oncology Group performance status (ECOG) 0 (219, 66%). Left-sided breast cancer (168, 51%) was slightly more common than right-sided breast cancer. Histology was most commonly invasive ductal carcinoma (283, 85%) and grades 1–2 (219, 66%). Estrogen receptor positivity was noted in 271 (82%) tumors, whereas human epidermal growth factor receptor 2 (HER-2) positivity was found in 74 (23%) patients. Pathologic tumor stage was T1–T2 in 309 (94%) patients. Eighty-seven (29%) patients were node-positive and only four (1%) patients had a metastasis at the time of initial diagnosis. Metastatic sites of disease in the four patients include liver (two), posterior mediastinal lymph node (one), and bone (one). All patients with metastatic disease received neoadjuvant chemotherapy and/or HER-2–directed therapy followed by modified radical mastectomy, followed by chest wall and regional nodal irradiation. These patients were then treated with adjuvant therapy such as endocrine therapy and or HER-2–directed therapy such as pertuzumab and trastuzumab. Of the two patients with liver metastases, one underwent partial hepatic resection. The other patient developed a clinical and radiographic response following neoadjuvant chemotherapy and did not undergo hepatic resection. The patient with bone metastases received palliative radiation 30 Gy in 10 fractions to multiple sites of bony metastasis. The patient initially presenting with a posterior mediastinal lymph node developed an excellent response to neoadjuvant chemotherapy and continued with HER-2–directed therapy after adjuvant radiotherapy.

**Table 1:** Patient and treatment characteristics by fractionation.

	All (N = 331)	Hypofractionation (N = 246)	Standard fractionation(N = 85)	p-value
**Age at RT Start**				<0.001^1^
Mean (SD)	58.6 (11.2)	59.8 (10.1)	55.0 (13.2)	
Median	60.0	62.0	54.0	
Range	29.0–87.0	31.0–87.0	29.0–82.0	
**Race**				0.378^2^
White	312 (95.1%)	233 (95.9%)	79 (92.9%)	
Non-White	16 (4.9%)	10 (4.1%)	6 (7.1%)	
Missing	3	3	0	
**Ethnicity**				0.524^2^
Hispanic	32 (9.8%)	22 (9.1%)	10 (11.8%)	
Non-Hispanic	296 (90.2%)	221 (90.9%)	75 (88.2%)	
Missing	3	3	0	
**Height (cm)**				0.320^1^
Mean (SD)	164.4 (6.3)	164.2 (6.1)	165.0 (6.7)	
Median	164.0	164.0	165.0	
Range	148.0–183.0	148.0–181.1	150.0–183.0	
**Weight (kg)**				0.923^1^
Mean (SD)	75.0 (17.3)	74.9 (17.8)	75.1 (15.7)	
Median	71.2	71.2	71.2	
Range	45.7–147.6	45.7–147.6	49.0–118.0	
**BMI**				0.817^1^
Mean (SD)	27.8 (6.2)	27.8 (6.4)	27.6 (5.6)	
Median	26.8	26.8	26.7	
Range	17.3–50.7	17.3–50.7	19.2–43.9	
**ECOG**				<0.001^2^
0	219 (66.2%)	179 (72.8%)	40 (47.1%)	
1	108 (32.6%)	64 (26.0%)	44 (51.8%)	
2	4 (1.2%)	3 (1.2%)	1 (1.2%)	
**ECOG 1+**				<0.001^2^
Yes	112 (33.8%)	67 (27.2%)	45 (52.9%)	
No	219 (66.2%)	179 (72.8%)	40 (47.1%)	
**ECOG 2+**				1.000^2^
Yes	4 (1.2%)	3 (1.2%)	1 (1.2%)	
No	327 (98.8%)	243 (98.8%)	84 (98.8%)	
**ER**				0.035^2^
Positive	271 (81.9%)	208 (84.6%)	63 (74.1%)	
Negative	60 (18.1%)	38 (15.4%)	22 (25.9%)	
**PR**				0.182^2^
Positive	255 (77.0%)	194 (78.9%)	61 (71.8%)	
Negative	76 (23.0%)	52 (21.1%)	24 (28.2%)	
**HER2**				0.002^2^
Yes	33 (10.1%)	19 (7.8%)	14 (16.9%)	
No	219 (67.2%)	159 (65.4%)	60 (72.3%)	
Not reported	74 (22.7%)	65 (26.7%)	9 (10.8%)	
Missing	5	3	2	
**Triple-Negative**				0.066^2^
Yes	27 (8.3%)	16 (6.6%)	11 (13.3%)	
No	299 (91.7%)	227 (93.4%)	72 (86.7%)	
Missing	5	3	2	
**Laterality**				1.000^2^
Right	163 (49.2%)	121 (49.2%)	42 (49.4%)	
Left	168 (50.8%)	125 (50.8%)	43 (50.6%)	
**Surgery Type**				<0.001^1^
Lumpectomy	229 (93.1%)	49 (57.6%)	278 (84.0%)	
Mastectomy	17 (6.9%)	36 (42.4%)	53 (16.0%)	
**Reconstruction After Mastectomy**				<0.001^2^
Yes	26 (7.9%)	6 (2.4%)	20 (23.5%)	
No	304 (91.8%)	240 (97.6%)	64 (75.3%)	
Not reported	1 (0.3%)	0 (0.0%)	1 (1.2%)	
**Any Chemotherapy**				<0.001^2^
Yes	129 (39.0%)	71 (28.9%)	58 (68.2%)	
No	202 (61.0%)	175 (71.1%)	27 (31.8%)	
**Adjuvant Chemotherapy**				0.143^2^
Yes	49 (39.5%)	31 (45.6%)	18 (32.1%)	
No	75 (60.5%)	37 (54.4%)	38 (67.9%)	
Missing	207	178	29	
**Neoadjuvant Chemotherapy**				0.138^2^
Yes	77 (62.1%)	38 (55.9%)	39 (69.6%)	
No	47 (37.9%)	30 (44.1%)	17 (30.4%)	
Missing	207	178	29	
**Concurrent Chemo**				0.001^2^
Yes	39 (11.8%)	20 (8.1%)	19 (22.4%)	
No	292 (88.2%)	226 (91.9%)	66 (77.6%)	
**Anthracycline**				1.000^2^
Yes	14 (10.5%)	8 (10.8%)	6 (10.2%)	
No	119 (89.5%)	66 (89.2%)	53 (89.8%)	
Missing	198	172	26	
**Modality**				<0.001^2^
Proton	50 (15.1%)	24 (9.8%)	26 (30.6%)	
Photon	281 (84.9%)	222 (90.2%)	59 (69.4%)	
**Total Dose**				<0.001^1^
Mean (SD)	42.6 (4.3)	40.2 (0.5)	49.7 (1.5)	
Median	40.0	40.0	50.0	
Range	40.0–50.4	40.0–42.8	45.0–50.4	
**Total Fractions**				<0.001^1^
Mean (SD)	17.8 (4.8)	15.0 (0.2)	25.9 (1.5)	
Median	15.0	15.0	25.0	
Range	15.0–28.0	15.0–17.0	22.0–28.0	
**Dose per Fraction**				<0.001^1^
Mean (SD)	2.5 (0.3)	2.7 (0.0)	1.9 (0.1)	
Median	2.7	2.7	2.0	
Range	1.8–2.7	2.5–2.7	1.8–2.1	
**Boost**				0.198^2^
Yes	131 (39.6%)	92 (37.4%)	39 (45.9%)	
No	200 (60.4%)	154 (62.6%)	46 (54.1%)	
**Boost Dose**				0.787^1^
Mean (SD)	9.9 (0.6)	9.9 (0.5)	9.9 (0.7)	
Median	10.0	10.0	10.0	
Range	5.4–10.5	7.5–10.0	5.4–10.5	
**Boost Fractions**				0.050^1^
Mean (SD)	4.8 (0.5)	4.7 (0.5)	4.9 (0.4)	
Median	5.0	5.0	5.0	
Range	3.0–5.0	3.0–5.0	3.0–5.0	
**Target**				<0.001^2^
WBRT	254 (77.0%)	225 (91.5%)	29 (34.5%)	
CW	71 (21.5%)	17 (6.9%)	54 (64.3%)	
PBI	5 (1.5%)	4 (1.6%)	1 (1.2%)	
Missing	1	0	1	
**Lymph Node Coverage**				<0.001^2^
Yes	48 (35.8%)	19 (18.1%)	29 (100.0%)	
No	86 (64.2%)	86 (81.9%)	0 (0.0%)	
Missing	197	141	56	
**Subsequent Cancer Treatment**				1.000^2^
Yes	168 (68.6%)	128 (68.4%)	40 (69.0%)	
No	77 (31.4%)	59 (31.6%)	18 (31.0%)	
Missing	86	59	27	
**Bolus**				<0.001^1^
Mean (SD)	0.2 (0.4)	0.1 (0.2)	0.6 (0.5)	
Median	0.0	0.0	1.0	
Range	0.0–1.0	0.0–1.0	0.0–1.0	
**Histology**				0.983^2^
Invasive ductal	283 (85.5%)	209 (85.0%)	74 (87.1%)	
Invasive lobular	13 (3.9%)	10 (4.1%)	3 (3.5%)	
Mixed ductal and lobular	5 (1.5%)	4 (1.6%)	1 (1.2%)	
Other	30 (9.1%)	23 (9.3%)	7 (8.2%)	
**Ductal Only**				0.723^2^
Yes	283 (85.5%)	209 (85.0%)	74 (87.1%)	
No	48 (14.5%)	37 (15.0%)	11 (12.9%)	
**pT**				<0.001^2^
T0	25 (7.6%)	15 (6.1%)	10 (11.8%)	
Tis (DCIS)	52 (15.8%)	45 (18.4%)	7 (8.2%)	
T1	169 (51.2%)	146 (59.6%)	23 (27.1%)	
T2	63 (19.1%)	31 (12.7%)	32 (37.6%)	
T3	21 (6.4%)	8 (3.3%)	13 (15.3%)	
Missing	1	1	0	
**pT 3+**				<0.001^2^
Yes	21 (6.4%)	8 (3.3%)	13 (15.3%)	
No	309 (93.6%)	237 (96.7%)	72 (84.7%)	
Missing	1	1	0	
**DCIS**				0.026^2^
Yes	52 (15.8%)	45 (18.4%)	7 (8.2%)	
No	278 (84.2%)	200 (81.6%)	78 (91.8%)	
Missing	1	1	0	
**pN**				<0.001^2^
N0	211 (70.8%)	187 (86.6%)	24 (29.3%)	
N1mi	5 (1.7%)	3 (1.4%)	2 (2.4%)	
N1	50 (16.8%)	17 (7.9%)	33 (40.2%)	
N2	24 (8.1%)	8 (3.7%)	16 (19.5%)	
N3	8 (2.7%)	1 (0.5%)	7 (8.5%)	
Missing	33	30	3	
**Node Positive**				<0.001^2^
Yes	87 (29.2%)	29 (13.4%)	58 (70.7%)	
No	211 (70.8%)	187 (86.6%)	24 (29.3%)	
Missing	33	30	3	
**cM**				0.273^2^
M0	327 (98.8%)	244 (99.2%)	83 (97.6%)	
M1	4 (1.2%)	2 (0.8%)	2 (2.4%)	
**Grade**				0.024^2^
1	71 (21.5%)	61 (24.9%)	10 (11.8%)	
2	148 (44.8%)	108 (44.1%)	40 (47.1%)	
3	111 (33.6%)	76 (31.0%)	35 (41.2%)	
Missing	1	1	0	
**SLNB**				<0.001^2^
Yes	223 (67.4%)	181 (73.6%)	42 (49.4%)	
No	108 (32.6%)	65 (26.4%)	43 (50.6%)	
**ALND**				<0.001^2^
Yes	71 (21.5%)	17 (6.9%)	54 (63.5%)	
No	260 (78.5%)	229 (93.1%)	31 (36.5%)	
**Any Grade 2+ PRO**				0.030^2^
Yes	247 (74.6%)	176 (71.5%)	71 (83.5%)	
No	84 (25.4%)	70 (28.5%)	14 (16.5%)	
**Any Grade 3+ PRO**				<0.001^2^
Yes	106 (32.0%)	65 (26.4%)	41 (48.2%)	
No	225 (68.0%)	181 (73.6%)	44 (51.8%)	

^1^Linear model ANOVA.

^2^Fisher’s exact test for count data.

### Treatment characteristics

**Table 1** also highlights the treatment characteristics in this patient cohort. Breast-conserving surgery, which consisted of lumpectomy, occurred in 202 (82%) and 47 (55%) who underwent adjuvant hypofractionated radiotherapy and conventionally fractionated radiotherapy, respectively. Mastectomy was performed in 53 (16%) patients, and lumpectomy was performed in 278 (84%). CF was more likely to be performed following mastectomy, with 17 (7%) HF vs. 31 (37%) CF (p < 0.001). A re-excision of a positive margin was performed in 34 (10%). Reconstruction occurred in 6 (2%) HF and 20 (24%) CF (p < 0.001). Sentinel lymph node biopsy (SLNB) occurred in 223 patients (67%) total, which consisted of 181 (55%) HF and 42 (12%) CF. CF was also more likely to be performed after axillary lymph node dissection with 17 (7%) undergoing HF and 54 (64%) undergoing CF (p < 0.001). A total of 37 (11%) patients did not undergo SLNB or ALND. Chemotherapy was administered—either in the neoadjuvant or adjuvant setting—to 129 (39%) patients: 71 (21.4%) HF and 58 (18%) CF.

Photons were utilized in 281 (85%) and protons were utilized in 50 (15%) patients. Photons were delivered in 281 (85%), and protons were delivered in 50 patients (15%). Protons were more likely to be delivered in patients receiving CF (26/85; 31%) vs. HF (24/246; 10%). Target coverage was to the whole breast in 254 (77%) patients, chest wall in 71 (22%) patients, and partial breast in five (1%) patients. Whole breast radiation was more likely to be HF (225/246; 92%) than CF (29/85; 35). Patients undergoing radiation following mastectomy were more likely to receive CF (54/85; 64.3%) compared with HF (17/246; 91.5%).

Forty-eight (36%) patients receiving lymph node coverage, with patients receiving CF more likely to have axillary lymph nodes included in the target volume than HF (34.1% vs. 7.7%, p < 0.001). The median dose/fractionation was 40.05 Gy/15 fractions for the HF group and 50 Gy/25 fractions for the CF group. The median boost dose/fractionation was 10 Gy/5 fractions for both groups. Forty-eight (36%) patients had lymph node coverage included in radiation treatment: 19(8%) HF and 29 CF (34%).

### Patient-reported adverse events

Two hundred forty-seven patients (75%) experienced any PRO-CTCAE grade ≥2 (**Table 2**), and 92 (28%) patients experienced any PRO-CTCAE grade ≥3 toxicity (**Table 3**). **Table 4** highlights the individual grade ≥2 PRO-CTCAE toxicities. Grade 2+ PROs were experienced by 176/246 (72%) HF and 71/85 (84%), with CF correlating with higher rates of grade 2+ PRO-CTCAE (p < 0.03). When evaluating modality, there was a statistically significant difference (p = 0.021) in grade ≥2 PRO-CTCAE in patients treated with protons (44/50; 88%) vs. photons (203/281; 72%). However, there was no difference in PRO-CTCAE grade ≥2 skin toxicity between protons and photons (14.2% vs. 11.5%). In addition, there was no difference in grade >3 PRO-CTCAE between protons (21/50; 42%) and photons (85/281; 30%) (p = 0.10). Skin toxicity (204, 76%) and insomnia (133, 40%) were the two most common PRO-CTCAEs graded ≥2. CF also correlated with higher rates of grade 3+ PRO, with 65/246 (26%) HF and 41/85 (48%) experiencing grade 3+ PRO (p < 0.001). The most common PRO-CTCAE ≥3 was skin toxicity (87, 33%) and concentration (92, 28%). When analyzing individual items by CF versus HF, CF was associated with increased risk of grade ≥2 skin toxicity (p = 0.04) and swallowing (p < 0.01), whereas HF was associated with an increased risk of grade ≥2 insomnia (p = 0.04) (**Table 2**). CF was also found to be associated with increased risk of grade ≥3 skin toxicity, swallowing, and nausea (all p < 0.01). When evaluating items most likely related to radiation (anxiety, cough, severity of breathing, interference with breathing, nausea, skin burns, and swallowing), CF was significantly associated with increased risk of PRO-CTCAE grade ≥3 (OR 2.96, p < 0.01).

**Table 2:** Patient characteristics by patients with any grade 2+ PRO CTCAE.

		Any Grade 2+ PRO CTCAE		
	All (N = 331)	Yes (N = 247)	No (N = 84)	p-value
**Modality**				0.021^1^
Proton	50 (15.1%)	44 (17.8%)	6 (7.1%)	
Photon	281 (84.9%)	203 (82.2%)	78 (92.9%)	
**Age at RT Start**				0.465^2^
Mean (SD)	58.6 (11.2)	58.3 (11.6)	59.3 (9.9)	
Median	60.0	59.0	60.5	
Range	29.0–87.0	29.0–87.0	34.0–78.0	
**Race**				1.000^1^
White	312 (95.1%)	232 (95.1%)	80 (95.2%)	
Non-White	16 (4.9%)	12 (4.9%)	4 (4.8%)	
Missing	3	3	0	
**Ethnicity**				0.400^1^
Hispanic	32 (9.8%)	22 (9.0%)	10 (12.0%)	
Non-Hispanic	296 (90.2%)	223 (91.0%)	73 (88.0%)	
Missing	3	2	1	
**Height (cm)**				0.471^2^
Mean (SD)	164.4 (6.3)	164.2 (6.3)	164.8 (6.3)	
Median	164.0	164.0	165.0	
Range	148.0–183.0	148.0–183.0	150.0–181.1	
**Weight (kg)**				0.814^2^
Mean (SD)	75.0 (17.3)	74.9 (16.0)	75.4 (20.7)	
Median	71.2	71.3	71.2	
Range	45.7–147.6	49.0–129.9	45.7–147.6	
**BMI**				0.771^2^
Mean (SD)	27.8 (6.2)	27.8 (6.1)	27.6 (6.6)	
Median	26.8	26.9	26.3	
Range	17.3–50.7	17.3–50.7	18.6–48.8	
**ECOG**				0.009^1^
0	219 (66.2%)	152 (61.5%)	67 (79.8%)	
1	108 (32.6%)	91 (36.8%)	17 (20.2%)	
2	4 (1.2%)	4 (1.6%)	0 (0.0%)	
**ECOG 1+**				0.002^1^
Yes	112 (33.8%)	95 (38.5%)	17 (20.2%)	
No	219 (66.2%)	152 (61.5%)	67 (79.8%)	
**ECOG 2+**				0.576^1^
Yes	4 (1.2%)	4 (1.6%)	0 (0.0%)	
No	327 (98.8%)	243 (98.4%)	84 (100.0%)	
**ER**				1.000^1^
Positive	271 (81.9%)	202 (81.8%)	69 (82.1%)	
Negative	60 (18.1%)	45 (18.2%)	15 (17.9%)	
**PR**				0.765^1^
Positive	255 (77.0%)	189 (76.5%)	66 (78.6%)	
Negative	76 (23.0%)	58 (23.5%)	18 (21.4%)	
**HER2**				0.365^1^
Yes	33 (10.1%)	22 (9.1%)	11 (13.3%)	
No	219 (67.2%)	168 (69.1%)	51 (61.4%)	
Not reported	74 (22.7%)	53 (21.8%)	21 (25.3%)	
Missing	5	4	1	
**Triple-Negative**				0.646^1^
Yes	27 (8.3%)	19 (7.8%)	8 (9.6%)	
No	299 (91.7%)	224 (92.2%)	75 (90.4%)	
Missing	5	4	1	
**Laterality**				0.102^1^
Right	163 (49.2%)	115 (46.6%)	48 (57.1%)	
Left	168 (50.8%)	132 (53.4%)	36 (42.9%)	
**Type of Surgery**				0.084^1^
Lumpectomy	278 (84.0%)	202 (81.8%)	76 (90.5%)	
Mastectomy	53 (16.0%)	45 (18.2%)	8 (9.5%)	
**Type of Reconstruction**				0.442^1^
Yes	26 (7.9%)	22 (8.9%)	4 (4.8%)	
No	304 (91.8%)	224 (90.7%)	80 (95.2%)	
Not reported	1 (0.3%)	1 (0.4%)	0 (0.0%)	
**Any Chemotherapy**				0.366^1^
Yes	129 (39.0%)	100 (40.5%)	29 (34.5%)	
No	202 (61.0%)	147 (59.5%)	55 (65.5%)	
**Adjuvant Chemotherapy**				0.665^1^
Yes	49 (39.5%)	39 (41.1%)	10 (34.5%)	
No	75 (60.5%)	56 (58.9%)	19 (65.5%)	
Missing	207	152	55	
**Neoadjuvant Chemotherapy**				0.512^1^
Yes	77 (62.1%)	57 (60.0%)	20 (69.0%)	
No	47 (37.9%)	38 (40.0%)	9 (31.0%)	
Missing	207	152	55	
**Concurrent Chemo**				0.696^1^
Yes	39 (11.8%)	28 (11.3%)	11 (13.1%)	
No	292 (88.2%)	219 (88.7%)	73 (86.9%)	
**Anthracycline**				0.519^1^
Yes	14 (10.5%)	12 (11.8%)	2 (6.5%)	
No	119 (89.5%)	90 (88.2%)	29 (93.5%)	
Missing	198	145	53	

^1^Fisher’s exact test for count data.

^2^Linear model ANOVA.

**Table 3:** Patient characteristics by patients with any grade 3+ PRO CTCAE.

		Any Grade 3+ PRO CTCAE		
	All (N = 331)	Yes (N = 106)	No (N = 225)	p-value
**Modality**				0.104^1^
Proton	50 (15.1%)	21 (19.8%)	29 (12.9%)	
Photon	281 (84.9%)	85 (80.2%)	196 (87.1%)	
**Age at RT Start**				0.559^2^
Mean (SD)	58.6 (11.2)	58.0 (11.5)	58.8 (11.0)	
Median	60.0	57.5	60.0	
Range	29.0–87.0	32.0–82.0	29.0–87.0	
**Race**				0.287^1^
White	312 (95.1%)	102 (97.1%)	210 (94.2%)	
Non-White	16 (4.9%)	3 (2.9%)	13 (5.8%)	
Missing	3	1	2	
**Ethnicity**				0.693^1^
Hispanic	32 (9.8%)	9 (8.5%)	23 (10.4%)	
Non-Hispanic	296 (90.2%)	97 (91.5%)	199 (89.6%)	
Missing	3	0	3	
**Height (cm)**				0.817^2^
Mean (SD)	164.4 (6.3)	164.5 (6.9)	164.3 (6.0)	
Median	164.0	163.5	164.1	
Range	148.0–183.0	150.0–183.0	148.0–181.1	
**Weight (kg)**				0.799^2^
Mean (SD)	75.0 (17.3)	75.3 (16.7)	74.8 (17.6)	
Median	71.2	71.0	71.3	
Range	45.7–147.6	49.0–118.0	45.7–147.6	
**BMI**				0.839^2^
Mean (SD)	27.8 (6.2)	27.9 (6.0)	27.7 (6.3)	
Median	26.8	26.7	26.9	
Range	17.3–50.7	18.7–47.4	17.3–50.7	
**ECOG**				0.003^1^
0	219 (66.2%)	58 (54.7%)	161 (71.6%)	
1	108 (32.6%)	45 (42.5%)	63 (28.0%)	
2	4 (1.2%)	3 (2.8%)	1 (0.4%)	
**ECOG 1+**				0.003^1^
Yes	112 (33.8%)	48 (45.3%)	64 (28.4%)	
No	219 (66.2%)	58 (54.7%)	161 (71.6%)	
**ECOG 2+**				0.098^1^
Yes	4 (1.2%)	3 (2.8%)	1 (0.4%)	
No	327 (98.8%)	103 (97.2%)	224 (99.6%)	
**ER**				1.000^1^
Positive	271 (81.9%)	87 (82.1%)	184 (81.8%)	
Negative	60 (18.1%)	19 (17.9%)	41 (18.2%)	
**PR**				0.675^1^
Positive	255 (77.0%)	80 (75.5%)	175 (77.8%)	
Negative	76 (23.0%)	26 (24.5%)	50 (22.2%)	
**HER2**				0.556^1^
Yes	33 (10.1%)	9 (8.6%)	24 (10.9%)	
No	219 (67.2%)	75 (71.4%)	144 (65.2%)	
Not reported	74 (22.7%)	21 (20.0%)	53 (24.0%)	
Missing	5	1	4	
**Triple-Negative**				1.000^1^
Yes	27 (8.3%)	9 (8.6%)	18 (8.1%)	
No	299 (91.7%)	96 (91.4%)	203 (91.9%)	
Missing	5	1	4	
**Laterality**				0.814^1^
Right	163 (49.2%)	51 (48.1%)	112 (49.8%)	
Left	168 (50.8%)	55 (51.9%)	113 (50.2%)	
**Type of Surgery**				0.006^1^
Lumpectomy	278 (84.0%)	80 (75.5%)	198 (88.0%)	
Mastectomy	53 (16.0%)	26 (24.5%)	27 (12.0%)	
**Type of Reconstruction**				0.508^1^
Yes	26 (7.9%)	11 (10.4%)	15 (6.7%)	
No	304 (91.8%)	95 (89.6%)	209 (92.9%)	
Not reported	1 (0.3%)	0 (0.0%)	1 (0.4%)	
**Any Chemotherapy**				0.117^1^
Yes	129 (39.0%)	48 (45.3%)	81 (36.0%)	
No	202 (61.0%)	58 (54.7%)	144 (64.0%)	
**Adjuvant Chemotherapy**				0.449^1^
Yes	49 (39.5%)	21 (44.7%)	28 (36.4%)	
No	75 (60.5%)	26 (55.3%)	49 (63.6%)	
Missing	207	59	148	
**Neoadjuvant Chemotherapy**				0.255^1^
Yes	77 (62.1%)	26 (55.3%)	51 (66.2%)	
No	47 (37.9%)	21 (44.7%)	26 (33.8%)	
Missing	207	59	148	
**Concurrent Chemo**				0.587^1^
Yes	39 (11.8%)	14 (13.2%)	25 (11.1%)	
No	292 (88.2%)	92 (86.8%)	200 (88.9%)	
**Anthracycline**				0.571^1^
Yes	14 (10.5%)	4 (8.2%)	10 (11.9%)	
No	119 (89.5%)	45 (91.8%)	74 (88.1%)	
Missing	198	57	141	

^1^Fisher’s exact test for count data.

^2^Linear model ANOVA.

**Table 4:** PRO-CTCAE ≥ 2 and ≥3 by individual items.

Question	PROCTACE ≥2	Odds Ratio (Standard Fractionation)	p-value
Anxiety	108 (32%)		
Appetite	27 (8%)		
Concentration	104 (31%)		
Cough	98 (31%)		
Insomnia	133 (40%)	0.57 (0.34–0.97)	0.04
Interfere with Breath	30 (9%)		
Nausea	52 (16%)		
Sadness	98 (30%)		
Severity of breath	97 (30%)		
Skin	204 (76%)	2.11 (1.03–4.31)	0.04
Swallowing	54 (18%)	3.14 (1.70–5.81)	<0.01
Tingling	40 (12%)		
	PROCTCAE ≥3		
Anxiety	51 (15%)		
Appetite	9 (3%)		
Concentration	92 (28%)		
Cough	33 (10%)		
Insomnia	91 (28%)		
Interfere with Breath	13 (4%)		
Nausea	19 (6%)	4.39 (1.70–11.32)	<0.01
Sadness	83 (25%)		
Severity of breath	60 (19%)		
Skin	87 (33%)	5.58 (3.13–9.95)	<0.01
Swallowing	20 (7%)	4.86 (1.91–12.40)	<0.01
Tingling	18 (6%)		

Univariate and multivariate analyses of clinical and treatment variables for any PRO-CTCAE ≥2 and ≥3 were performed (**Table 5**). The following variables were significantly associated with an increase in any PRO-CTCAE ≥2 after univariate analysis: ECOG ≥1, proton therapy (vs. photon), and chest wall radiation (vs. whole breast) (**Table 3**). HF was associated with decrease in any PRO-CTCAE ≥2. However, only ECOG ≥1 remained significant after multivariate analysis (OR 2.21, p = 0.01). When analyzing any PRO-CTCAE ≥3, ECOG ≥1, node positivity, and total dose were all univariately significant for increased risk of analyzing any PRO-CTCAE ≥3. HF was protective against PRO-CTCAE ≥3 (**Table 3**). In the multivariate model, ECOG ≥1 (OR 1.78, p = 0.02) and HF (OR 0.48, p < 0.01) remained significant.

**Table 5:** Univariate and multivariate analysis for any PRO-CTCAE ≥2 and ≥3.

UVA for any PROCTCAE ≥ 2	Odds Ratio	p-value
ECOG ≥1	2.46 (1.36–4.44)	<0.01
Proton	2.82 (1.14–6.87)	0.02
Chest wall (vs. breast)	2.41 (1.17–4.97)	0.05
Hypofractionation	0.51 (0.27–0.96)	0.04
MVA for any PROCTCAE ≥ 2		
ECOG ≥1	2.21 (1.21–4.06)	0.01
Proton		0.06
Chest wall		0.22
Hypofractionation		0.28
UVA for any PROCTCAE ≥ 3		
ECOG ≥1	2.08 (1.29–3.36)	<0.01
N+	1.82 (1.08–3.05)	0.02
Hypofractionation	0.4 (0.24–0.67)	<0.01
Total dose	1.09 (1.03–1.15)	<0.01
MVA for any PROCTCAE ≥ 3		
ECOG ≥1	1.78 (1.08–2.92)	0.02
N+		0.68
Hypofractionation	0.48 (0.28–0.82)	<0.01
Total dose		0.2

### Physician reported adverse events

Per physician reporting, a grade ≥2 adverse event was found in 114 patients: 52 (21%) HF and 62/85 (73%) CF. Radiation dermatitis was the most common adverse event reported with grade 2+ RT dermatitis occurring in 37 (72.5%) HF and 57 (70.4%) CF. Grade 3 radiation dermatitis occurred in four patients receiving CF, whereas there was none in the HF group.

## Discussion

Our study reports one of the first clinical experiences utilizing multiple PRO-CTCAE items for patients with breast cancer undergoing radiation therapy with curative intent. Moderate (≥2) and severe (≥3) PRO-CTCAE were commonly experienced in our patient cohort. Compared with CF, patients undergoing HF were associated with a significant decrease in any PRO-CTCAE ≥3 after multivariate analysis.

This is one of the largest series for a single cancer type reporting on multiple PRO-CTCAE items. Previous studies have incorporated multiple disease sites, other patient-reported outcome measures, or only focused on one PRO-CTCAE item. A Japanese study of over 600 patients with breast cancer analyzed a PRO-CTCAE for lymphedema and found it weakly correlated with arm circumference and bioimpedance (13). A smaller American study on patients undergoing APBI reported skin toxicity using PRO-CTCAE with 31% of patients reporting mild to moderate skin toxicity at the end of radiotherapy and only 15% of patients 12 months post-radiation (14). Similarly, in our study, 33% of patients experienced a skin PRO-CTCAE ≥2 at any time point, supporting the use of PRO-CTCAE across institutions.

Skin toxicity has been a primary concern for both patients and radiation oncologists. Publications have reported physician assessed CTCAE radiation dermatitis ≥2 ranging from 30% to 60%, depending on risk factors including treatment modality and dose fractionation (15–17). Regarding the use proton therapy as adjuvant radiotherapy following breast-conserving surgery or mastectomy, rates of grade 2 dermatitis may be high (18, 19). Cuaron et al. reported a grade 2+ dermatitis rate of 71% to 75% in 30 patients receiving postoperative proton radiation for locally advanced breast cancer (19). In addition, the rate of moist desquamation was 28.6% (19). Similarly, the physician reported that adverse event rate of radiation dermatitis was 72.5% in the HF group and 70.4% in the CF group. Across the entire cohort, the PRO CTCAE grade >2 rate of skin toxicity was 76%. This demonstrates that the patient experience of skin toxicity may be more severe than physician reported toxicity. Because PRO-CTCAE is specific to the individual patient, employing patient-reported toxicity may help clinicians identify and treat radiation-related adverse events using a more individualized approach.

HF has been established as a standard of care radiation treatment option in the breast-conserving setting and is gaining more support in the post-mastectomy setting (20–22). Compared with CF, HF has been shown to be associated with decreased physician-reported radiation dermatitis, breast pain, and hyperpigmentation (16, 23). Patient-reported outcomes and quality-of-life measures—including lack of energy, trouble meeting family needs, breast pain, swelling bother, and fatigue—have also been shown to be improved with HF (16, 17). This is consistent with the current analysis, in which HF was significantly associated with decreased risk of any PRO-CTCAE ≥3 in the multivariate model. In addition to delivering less radiation over a shorter amount of time, HF was more likely to be delivered following breast-conserving surgery for early-stage disease and not include axillary lymph nodes in the target volume. These could be contributory reasons why HF is associated with a significantly decreased risk of grade ≥3 individual items such as skin toxicity.

This study is limited by its observational nature. One of the benefits of the PRO-CTCAE is its implementation as a standardized approach to patient-reported outcomes in clinical trials. There may also be selection bias in the patients who choose to complete these surveys (i.e., patients who experience toxicity may be more likely to complete their questionnaire). There was also variation in time points when patients completed their surveys, which can influence the presence or severity of adverse events recorded. Moreover, this study did not utilize the PRO-CTCAE to guide management that limits its usefulness in the clinic. PRO-CTCAE will likely have the most benefit when used as a supplement to physician reported CTCAEs in the recognition and management of radiation toxicities.

## Conclusion

Through routinely administered patient-reported outcome surveys in our department, we have analyzed PRO-CTCAE items for over 300 patients undergoing radiation therapy for breast cancer. Rates of PRO-CTCAE skin toxicity were like those historically reported by physicians, and, of note, HF was associated with a significantly decreased risk of grade ≥3 PRO-CTCAE. Further study is warranted in other cancer-specific settings, comparing PRO-CTCAEs with CTCAEs and finally clinical implementation of PRO-CTCAE.

## Data availability statement

The raw data supporting the conclusions of this article will be made available by the authors, without undue reservation.

## Ethics statement

The studies involving human participants were reviewed and approved by Mayo Clinic Institutional Review Board. The patients/participants provided their written informed consent to participate in this study.

## Author contributions

CV contributed to conception and design of the study. TD and MG performed the statistical analysis. BL, RB, and CT wrote sections of the manuscript. All authors contributed to manuscript revision, read, and approved the submitted version.

## Funding

This research did not receive any specific grant from funding agencies in the public, commercial, or not-for-profit sectors.

## Conflict of interest

The authors declare that the research was conducted in the absence of any commercial or financial relationships that could be construed as a potential conflict of interest.

## Publisher’s note

All claims expressed in this article are solely those of the authors and do not necessarily represent those of their affiliated organizations, or those of the publisher, the editors and the reviewers. Any product that may be evaluated in this article, or claim that may be made by its manufacturer, is not guaranteed or endorsed by the publisher.

## Abbreviations

APBI, accelerated partial breast irradiation; CTCAE, Common Terminology Criteria for Adverse Events; NCI, National Cancer Institute; OAR, organ at risk; PCG, Proton Collaborative Group; PRO-CTCAE, Patient-Reported Outcomes Version of the Common Terminology Criteria for Adverse Events; PROMIS-10, Patient-Reported Outcomes Measurement Information System 10.

## References

[1] Atkinson TM Li Y Coffey CW Sit L Shaw M Lavene D. Reliability of adverse symptom event reporting by clinicians. Qual Life Res (2012) 21(7):1159–64. doi: 10.1007/s11136-011-0031-4 PMC3633532 21984468

[2] Quinten C Maringwa J Gotay CC Martinelli F Coens C Reeve BB. Patient self-reports of symptoms and clinician ratings as predictors of overall cancer survival. J Natl Cancer Inst (2011) 103(24):1851–8. doi: 10.1093/jnci/djr485 PMC3243678 22157640

[3] Fromme EK Eilers KM Mori M Hsieh YC Beer TM. How accurate is clinician reporting of chemotherapy adverse effects? a comparison with patient-reported symptoms from the quality-of-Life questionnaire C30. J Clin Oncol (2004) 22(17):3485–90. doi: 10.1200/JCO.2004.03.025 15337796

[4] Basch E Reeve BB Mitchell SA Clauser SB Minasian LM Dueck AC. Development of the national cancer institute's patient-reported outcomes version of the common terminology criteria for adverse events (PRO-CTCAE). J Natl Cancer Inst (2014) 106(9):1–11. doi: 10.1093/jnci/dju244 PMC4200059 25265940

[5] Dueck AC Mendoza TR Mitchell SA Reeve BB Castro KM Rogak LJ. Validity and reliability of the US national cancer institute's patient-reported outcomes version of the common terminology criteria for adverse events (PRO-CTCAE). JAMA Oncol (2015) 1(8):1051–9. doi: 10.1001/jamaoncol.2015.2639 PMC4857599 26270597

[6] Sandler KA Mitchell SA Basch E Raldow AC Steinberg ML Sharif J. Content validity of anatomic site-specific patient-reported outcomes version of the common terminology criteria for adverse events (PRO-CTCAE) item sets for assessment of acute symptomatic toxicities in radiation oncology. Int J Radiat Oncol Biol Phys (2018) 102(1):44–52. doi: 10.1016/j.ijrobp.2018.04.048 30102201

[7] Albaba H Barnes TA Veitch Z Brown MC Shakik S Su S. Acceptability of routine evaluations using patient-reported outcomes of common terminology criteria for adverse events and other patient-reported symptom outcome tools in cancer outpatients: Princess Margaret cancer centre experience. Oncologist. (2019) 24(11):e1219–e27. doi: 10.1634/theoncologist.2018-0830 PMC6853088 31409744

[8] Basch E Pugh SL Dueck AC Mitchell SA Berk L Fogh S. Feasibility of patient reporting of symptomatic adverse events *via* the patient-reported outcomes version of the common terminology criteria for adverse events (PRO-CTCAE) in a chemoradiotherapy cooperative group multicenter clinical trial. Int J Radiat Oncol Biol Phys (2017) 98(2):409–18. doi: 10.1016/j.ijrobp.2017.02.002 PMC5557037 28463161

[9] Dueck AC Scher HI Bennett AV Mazza GL Thanarajasingam G Schwab G. Assessment of adverse events from the patient perspective in a phase 3 metastatic castration-resistant prostate cancer clinical trial. JAMA Oncol (2019) 6(2):1–11. doi: 10.1001/jamaoncol.2019.3332 PMC6764147 31556911

[10] Yeung AR Pugh SL Klopp AH Gil KM Wenzel L Westin SN. Improvement in patient-reported outcomes with intensity-modulated radiotherapy (RT) compared with standard RT: A report from the NRG oncology RTOG 1203 study. J Clin Oncol (2020) 38(15):1685–92. doi: 10.1200/JCO.19.02381 PMC7238486 32073955

[11] Hammert WC Calfee RP. Understanding PROMIS. J Handb Surg Am (2020) 45:650–4. doi: 10.1016/j.jhsa.2020.03.016 32370909

[12] Jones FJS Ezzeddine FL Herman ST Buchhalter J Fureman B Moura L. A feasibility assessment of functioning and quality-of-life patient-reported outcome measures in adult epilepsy clinics: A systematic review. Epilepsy Behav (2020) 102:106704. doi: 10.1016/j.yebeh.2019.106704 31816482

[13] Terada M Yoshimura A Sawaki M Hattori M Naomi G Kotani H. Patient-reported outcomes and objective assessments with arm measurement and bioimpedance analysis for lymphedema among breast cancer survivors. Breast Cancer Res Treat (2020) 179(1):91–100. doi: 10.1007/s10549-019-05443-1 31535321

[14] Mutter RW Jethwa KR Gonuguntla K Remmes NB Whitaker TJ Hieken TJ. 3 fraction pencil-beam scanning proton accelerated partial breast irradiation: early provider and patient reported outcomes of a novel regimen. Radiat Oncol (2019) 14(1):211. doi: 10.1186/s13014-019-1417-7 31752934 PMC6873533

[15] Pignol JP Olivotto I Rakovitch E Gardner S Sixel K Beckham W. A multicenter randomized trial of breast intensity-modulated radiation therapy to reduce acute radiation dermatitis. J Clin Oncol (2008) 26(13):2085–92. doi: 10.1200/JCO.2007.15.2488 18285602

[16] Shaitelman SF Schlembach PJ Arzu I Ballo M Bloom ES Buchholz D. Acute and short-term toxic effects of conventionally fractionated vs hypofractionated whole-breast irradiation: A randomized clinical trial. JAMA Oncol (2015) 1(7):931–41. doi: 10.1001/jamaoncol.2015.2666 PMC4635441 26247543

[17] Jagsi R Griffith KA Boike TP Walker E Nurushev T Grills IS. Differences in the acute toxic effects of breast radiotherapy by fractionation schedule: Comparative analysis of physician-assessed and patient-reported outcomes in a Large multicenter cohort. JAMA Oncol (2015) 1(7):918–30. doi: 10.1001/jamaoncol.2015.2590 26247417

[18] Verma V Shah C Mehta MP. Clinical outcomes and toxicity of proton radiotherapy for breast cancer. Clin Breast Cancer. (2016) 16(3):145–54. doi: 10.1016/j.clbc.2016.02.006 26995159

[19] Cuaron JJ Chon B Tsai H Goenka A DeBlois D Ho A. Early toxicity in patients treated with postoperative proton therapy for locally advanced breast cancer. Int J Radiat Oncol Biol Physics. (2015) 92(2):284–91. doi: 10.1016/j.ijrobp.2015.01.005 PMC4972493 25754632

[20] Haviland JS Owen JR Dewar JA Agrawal RK Barrett J Barrett-Lee PJ. The UK standardisation of breast radiotherapy (START) trials of radiotherapy hypofractionation for treatment of early breast cancer: 10-year follow-up results of two randomised controlled trials. Lancet Oncol (2013) 14(11):1086–94. doi: 10.1016/S1470-2045(13)70386-3 24055415

[21] Whelan T MacKenzie R Julian J Levine M Shelley W Grimard L. Randomized trial of breast irradiation schedules after lumpectomy for women with lymph node-negative breast cancer. J Natl Cancer Inst (2002) 94(15):1143–50. doi: 10.1093/jnci/94.15.1143 12165639

[22] Wang SL Fang H Song YW Wang WH Hu C Liu YP. Hypofractionated versus conventional fractionated postmastectomy radiotherapy for patients with high-risk breast cancer: a randomised, non-inferiority, open-label, phase 3 trial. Lancet Oncol (2019) 20(3):352–60. doi: 10.1016/S1470-2045(18)30813-1 30711522

[23] Parekh A Dholakia AD Zabranksy DJ Asrari F Camp M Habibi M. Predictors of radiation-induced acute skin toxicity in breast cancer at a single institution: Role of fractionation and treatment volume. Adv Radiat Oncol (2018) 3(1):8–15. doi: 10.1016/j.adro.2017.10.007 29556573 PMC5856985

